# Aurora A Kinase Inhibitor AKI603 Induces Cellular Senescence in Chronic Myeloid Leukemia Cells Harboring T315I Mutation

**DOI:** 10.1038/srep35533

**Published:** 2016-11-08

**Authors:** Le-Xun Wang, Jun-Dan Wang, Jia-Jie Chen, Bing Long, Ling-Ling Liu, Xi-Xiang Tu, Yu Luo, Yuan Hu, Dong-Jun Lin, Gui Lu, Zi-Jie Long, Quentin Liu

**Affiliations:** 1 Department of Hematology, The Third Affiliated Hospital, Sun Yat-sen University, 600 Tianhe Road, Guangzhou 510630, China; Institute of Hematology, Sun Yat-sen University, Guangzhou 510630, China; 2 Department of Cardiac Surgery II, The First Affiliated Hospital, Sun Yat-sen University, 58 Zhongshan 2 Road, Guangzhou 510080, China; 3 Institute of Cancer Stem Cell, Dalian Medical University, 9 West Section, Lvshun South Road, Dalian 116044, China; 4 Institute of Medicinal Chemistry, School of Pharmaceutical Sciences, Sun Yat-sen University, 132 Waihuan Road East, Guangzhou 510006, China; 5 Sun Yat-sen University Cancer Center; State Key Laboratory of Oncology in South China; Collaborative Innovation Center of Cancer Medicine, 651 Dongfeng East Road, Guangzhou 510060, China

## Abstract

The emergence of resistance to imatinib mediated by mutations in the BCR-ABL has become a major challenge in the treatment of chronic myeloid leukemia (CML). Alternative therapeutic strategies to override imatinib-resistant CML are urgently needed. In this study, we investigated the effect of AKI603, a novel small molecule inhibitor of Aurora kinase A (AurA) to overcome resistance mediated by BCR-ABL-T315I mutation. Our results showed that AKI603 exhibited strong anti-proliferative activity in leukemic cells. AKI603 inhibited cell proliferation and colony formation capacities in imatinib-resistant CML cells by inducing cell cycle arrest with polyploidy accumulation. Surprisingly, inhibition of AurA by AKI603 induced leukemia cell senescence in both BCR-ABL wild type and T315I mutation cells. Furthermore, the induction of senescence was associated with enhancing reactive oxygen species (ROS) level. Moreover, the anti-tumor effect of AKI603 was proved in the BALB/c nude mice KBM5-T315I xenograft model. Taken together, our data demonstrate that the small molecule AurA inhibitor AKI603 may be used to overcome drug resistance induced by BCR-ABL-T315I mutation in CML.

Chronic myeloid leukemia (CML) is a myeloproliferative disorder that accounts for 15% of adult leukemia^1^. This disease is characterized by Philadelphia chromosome, the t (9; 22) (q34; q11) reciprocal translocation, resulting in the expression of a fusion protein BCR-ABL^2^,^3^. BCR-ABL plays a central role in the pathogenesis of CML by activating multiple signal pathways^4^,^5^,^6^. Thus, BCR-ABL has been an important target for CML therapeutics. Although the development of imatinib, a tyrosine kinase inhibitor (TKI) has redefined the management of CML^7^, the resistance to imatinib occurs in 20~30% of CML patients and is commonly attributable to point mutations in the BCR-ABL kinase domain^8^,^9^. In more than 100 mutations of BCR-ABL, T315I mutation is one of the most common mutations, and accounts for about 20% of imatinib-resistant cases^10^. However, T315I mutation confers resistance to multiple TKIs^11^. Hence, novel compounds or strategies to override this challenging problem are urgently required for CML treatment.

The discovery that AurA was abnormally expressed in malignancies including leukemia prompted the development of agents that inhibited kinase activity^12^. Small molecule kinase inhibitors of AurA have attracted a great interest. For example, MK-0457 (VX-680), PHA-739358 and MLN8237 are being investigated in clinical trials^12^,^13^,^14^,^15^. MK-0457 effectively inhibited proliferation and growth of multiple tumor cell types including HL-60 cells^14^,^16^. Our and other studies suggested that AurA kinase activity was responsible for chemo-resistance and tumorigenic ability^16^,^17^. MLN8237, MK-0457 and related compound VE-465 exhibited promising results against leukemia cells expressing T315I mutant form of BCR-ABL *in vitro*, *in vivo* and in patients^18^,^19^,^20^. Those studies indicate that AurA inhibitors exhibit a desirable therapeutic index in resistance of CML to imatinib caused by the T315I mutation.

The aim of this study was to investigate the antineoplastic effects of the novel AurA small molecule inhibitor AKI603 in CML cells. AKI603 inhibited cell proliferation and induced senescence both in BCR-ABL wild-type and BCR-ABL-T315I mutant CML cells as well as in nude mouse xenograft models. The results revealed that AKI603 could efficiently overcome imatinib resistance of CML *in vitro* and *in vivo*.

## Results

### AKI603 extensively inhibits proliferation of leukemia cells

**Figure 1:**
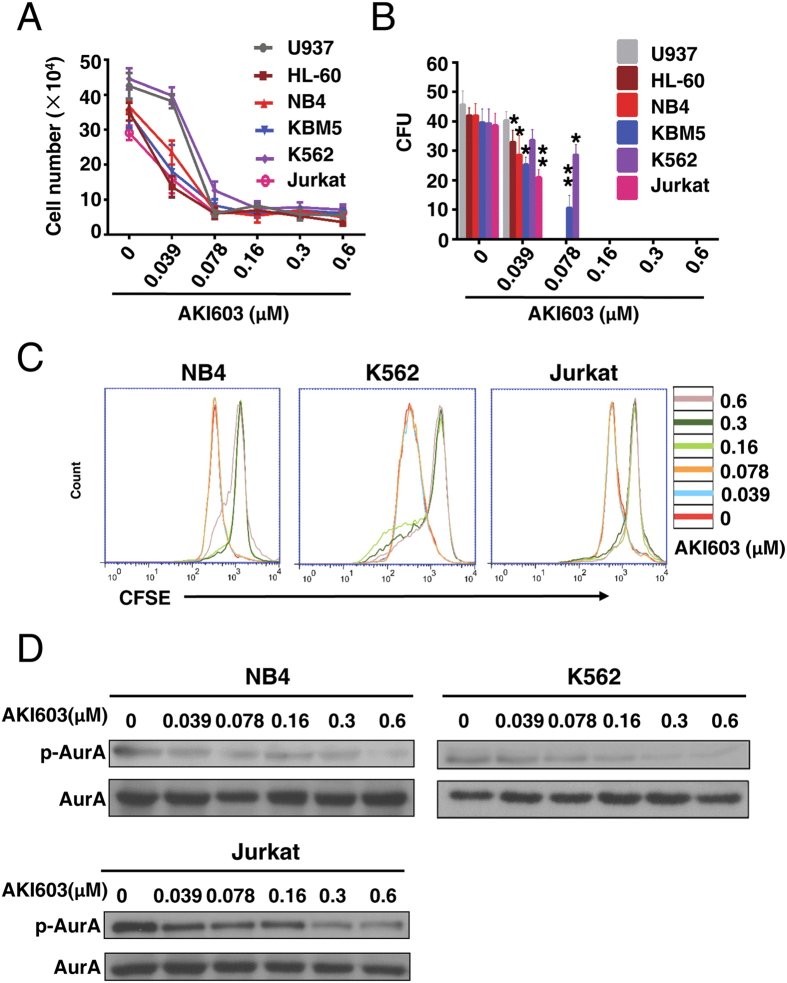
AKI603 extensively inhibits proliferation of leukemia cells. (**A**) Leukemia cells were treated with various concentrations of AKI603 (0 μM, 0.039 (0.0390125) μM, 0.078 (0.078125) μM, 0.16 (0.15625) μM, 0.3 (0.3125) μM, 0.6 (0.625) μM) for 48 h. Cell counting assay was performed. The mean values from three independent experiments are presented. (**B**) The colony formation of cells treated with AKI603 for 10 days were analyzed. The statistical analysis of the colony formation assay is shown (mean ± SD, *p < 0.05, **p < 0.01 vs. 0). (**C**) NB4, K562 and Jurkat cells were stained by CFSE and treated with various concentrations of AKI603 for 48 h. The levels of CFSE fluorescence were analyzed by flow cytometry. (**D**) NB4, K562 and Jurkat cells were treated with various concentrations of AKI603 for 48 h. The lysates were subjected for western blot analysis of p-AurA (Thr288) and AurA expression. The data are representative of three independent experiments.

We recently reported that AKI603 could inhibit the proliferation of breast cancer cells^21^. To evaluate the effect of AKI603 on proliferation of leukemic cells, six leukemic cell lines (AML (acute myelocytic leukemia): U937, HL-60 and NB4; CML: KBM5 and K562; ALL (acute lymphoblastic leukemia): Jurkat) were treated with various concentrations of AKI603 for 48 h, and the cell proliferation was determined by cell counting assay and CFSE (carboxyfluorescein diacetate succinimidyl ester) staining assay. As shown in Fig. 1A,C and Supplementary Fig. 1A, all the tested cell lines were inhibited by AKI603 treatment. We performed a colony formation assay in methylcellulose to test the long-term effect of AKI603 on leukemic cells. As shown in Fig. 1B, AKI603 potently decreased the number of colony units at concentration of 0.16 μM.

Next, we assessed the ability of AKI603 to inhibit the kinase activity of AurA in leukemic cells, by testing the phosphorylation of AurA Thr288 (p-AurA). As shown in Fig. 1D, AKI603 significantly inhibited the phosphorylation of AurA in NB4, K562, and Jurkat cell lines in a dose-dependent manner while the level of total AurA protein was not changed.

### AKI603 inhibits the proliferation and colony formation of imatinib resistant CML cells

**Figure 2:**
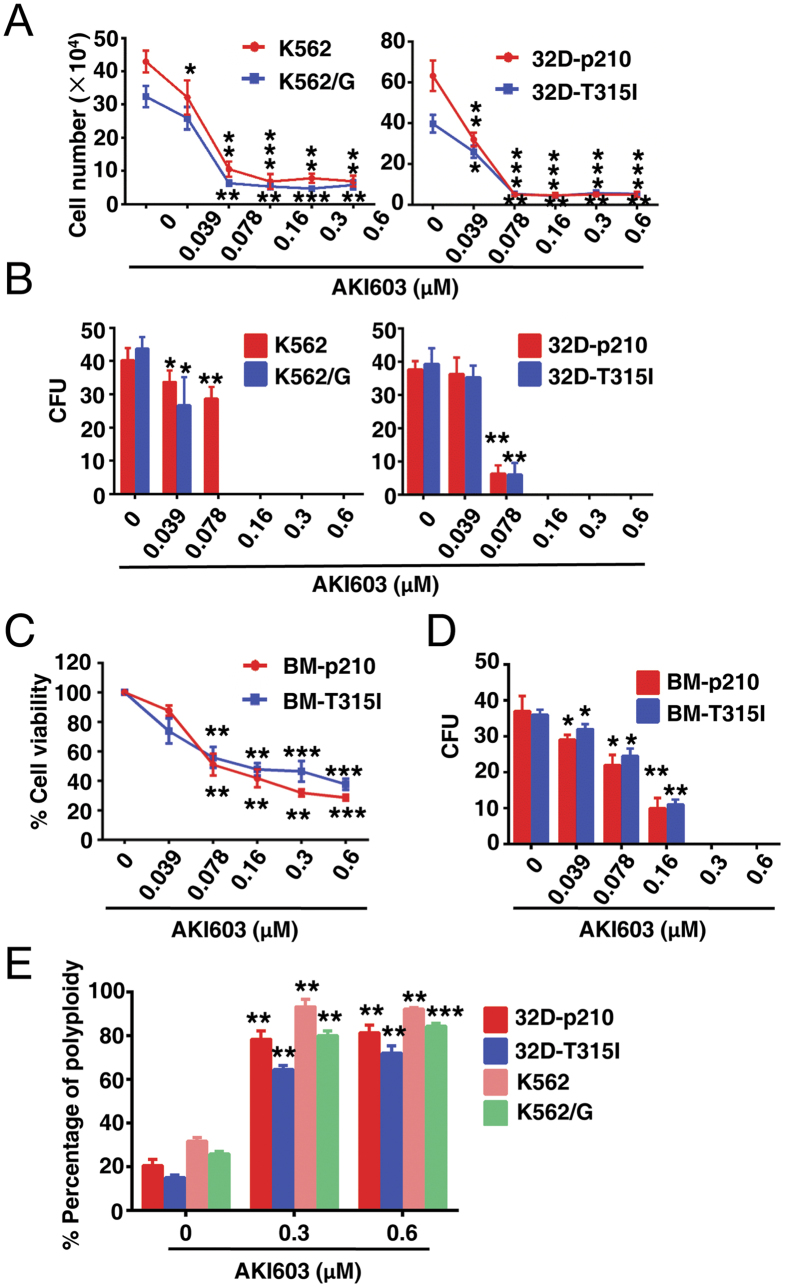
AKI603 inhibits proliferation and colony formation in imatinib resistant cells. (**A**) Imatinib sensitive cells (K562 and 32D-p210) and imatinib resistant cells (K552/G and 32D-T315I) were treated with various concentrations of AKI603 for 48 h. Cell counting assay was performed (mean ± SD, *p < 0.05, **p < 0.01, ***p < 0.001 vs. 0). (**B**) The colony formation of cells treated with AKI603 for 10 days were analyzed. The statistical analysis of the colony formation assay is shown (mean ± SD, *p < 0.05, **p < 0.01 vs. 0). (**C**) BM cells overexpressed with p210 or T315I were treated with various concentrations of AKI603 for 96 h. MTT assay was performed (mean ± SD, **p < 0.01, ***p < 0.001 vs. 0). (**D**) The colony formation of BM cells treated with AKI603 for 10 days were analyzed. The statistical analysis of the colony formation assay is shown (mean ± SD, *p < 0.05, **p < 0.01 vs. 0). (**E**) K562, K562/G, 32D-p210 and 32D-T315I cells were treated with various concentrations of AKI603 for 48 h and cell cycle was analyzed by flow cytometry (mean ± SD, **p < 0.01, ***p < 0.001 vs. 0).

K562, KBM5 are sensitive to imatinib treatment, whereas K562/G^22^ and KBM5-T315I^23^ cells are resistant to imatinib. To test the effect of AKI603 on proliferation of imatinib resistant CML cells, K562/G and KBM5-T315I cells were incubated for 48 h with different concentrations of AKI603. As shown in Fig. 2A and Supplementary Fig. 1B, all cell types were inhibited by 0.078 μM AKI603. To further validate this finding, we established a pair of 32D (32Dcl3) murine cell lines stably expressing wild-type (p210) or T315I-mutant (p210-T315I, T315I) BCR-ABL. 32D-p210 cells were sensitive to imatinib whereas 32D-T315I cells were resistant to imatinib (Supplementary Fig. S2A). Then we treated these two cells with various concentrations of AKI603 and found that the growth of cells (32D-p210 and 32D-T315I) was significantly inhibited, with IC50 values of 0.032 μM and 0.039 μM, respectively (Fig. 2A). In colony formation assay, the colony units of imatinib-resistant cells were inhibited in a dose-dependent manner (Fig. 2B).

In addition, we established a pair of BM (bone marrow) cells stably expressing p210 or p210-T315I of BCR-ABL. After 7 days of culture without cytokines, the survival cells of transfected BM was tested by Western blot for BCR-ABL expression (Supplementary Fig. S2B). Then we tested the sensitivity of those cells to imatinib and found that BM-p210 cells were sensitive whereas BM-T315I cells were resistant to imatinib (Supplementary Fig. S2C). The proliferation of both cells were significantly inhibited by AKI603 (Fig. 2C) and the colony unit was completely inhibited at the concentration of 0.3 μM (Fig. 2D).

Due to the crucial role of AurA in mitosis, the blocking effect of cell cycle by AKI603 was examined. AKI603 significantly induced polyploidization in K562, K562/G, 32D-p210 and 32D-T315I cells (Fig. 2E and Supplementary Fig. S3A). These results suggested that the proliferative inhibition induced by AKI603 could be associated with cell cycle blockage.

### Inhibition of AurA kinase by AKI603 results in cellular senescence

**Figure 3:**
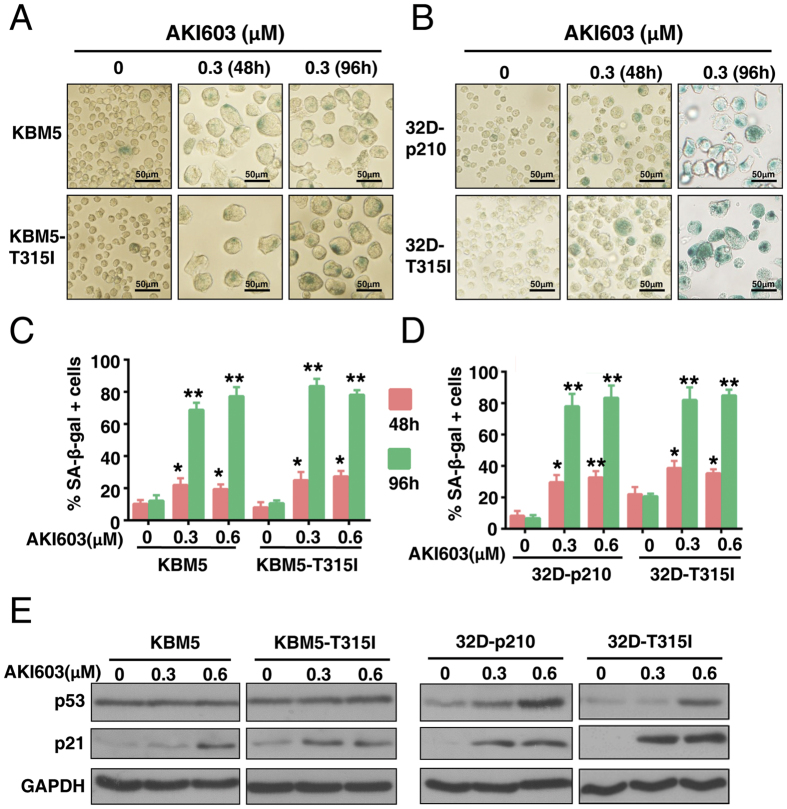
Inhibition of AurA kinase by AKI603 induces cellular senescence. (**A,B**) KBM5, KBM5-T315I, 32D-p210 and 32D-T315I cells were treated with 0.3 μM of AKI603 for 48 h or 96 h, and senescence was determined using SA-β-gal staining. (**C,D**) The statistical analysis of the SA-β-gal assay is shown. More than 300 cells per sample were counted to determine the percentage of senescent cells (mean ± SD, *p < 0.05, **p < 0.01 vs. 0). (**E**) KBM5, KBM5-T315I, 32D-p210 and 32D-T315I cells were treated with 0.3 μM and 0.6 μM of AKI603 for 96 h. The lysates were subjected to western blot to analyze the expression of p53 and p21. The data are representative of three independent experiments.

AKI603 could induce cell cycle arrest with polyploidy accumulation in K562, K562/G, 32D-p210 and 32D-T315I cells (Fig. 2E), but did not result in obvious apoptosis in 32D-p210 and 32D-T315I cells (Supplementary Fig. S3B). As apoptosis could not account for the significant reduction in cell number in T315I-mutant or wild-type BCR-ABL cell lines, we predicted that other processes were responsible for reduced cancer cell proliferation in response to AKI603 treatment. Indeed, after 96 h of treatment, we observed that the cellular size was greatly enlarged (Supplementary Fig. S3C), which is a characteristic of senescence. The morphological change we observed in leukemia cells was also consistent with the senescence phenotype described in AurA- or AurB (Aurora kinase B)-knockdown cells of solid tumors as well as leukemia cells^24^,^25^. To assess enlarged cellular size induced by AKI603 is associated with senescence, β-galactosidase activity was examined using SA-β-gal (senescence-associated β-galactosidase) assay. Results showed that β-galactosidase activity was enhanced in drug-treated KMB5, KBM5-T315I, 32D-p210 and 32D-T315I cells (Fig. 3A,B). After 96 h of treatment, the percentage of SA-β-gal positive cells of KBM5, KBM5-T315I, 32D-p210 and 32D-T315I at 0.3 μM was 68.8% ± 4.4%, 83.6% ± 5.6%, 78.7% ± 5.8% and 81.4% ± 6.2%, respectively (Fig. 3C,D).

To investigate the mechanism of this drug-induced senescence, we tested the expression of p21 in AKI603-treated cells by western blot. In response to drug treatment for 96 h, p21 was induced in p53 wt (32D-p210 and 32D-T315I)^26^ and p53-mutant (KBM5 and KBM5-T315I)^27^ cells (Fig. 3E). However, p53 was induced in 32D-p210 and 32D-T315I cells, but not in KBM5 and KBM5-T315I cells (Fig. 3E). These results suggested that p21 might be the essential regulator of AKI603-induced senescence independent of p53.

### Induction senescence of AKI603 is partially via enhancing ROS generation

**Figure 4:**
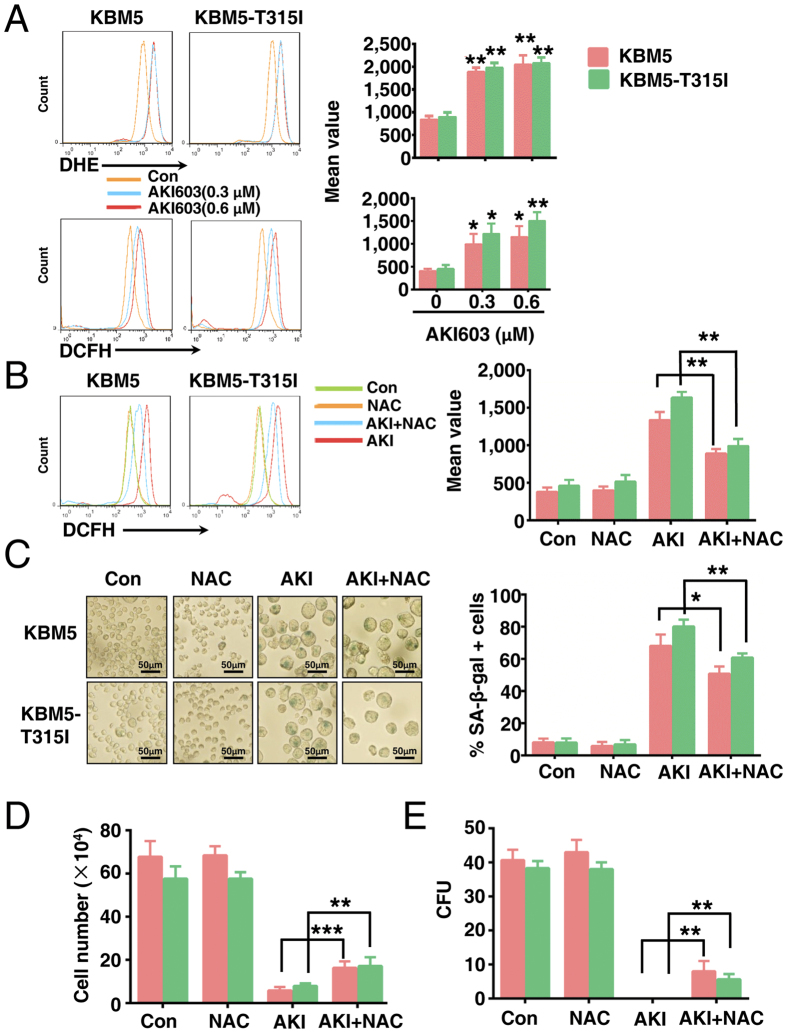
Induction of senescence by AKI603 is partially via enhancing ROS level. (**A**) KBM5 and KBM5-T315I cells were treated with indicated concentration of AKI603 for 96 h. The levels of DCFH and DHE fluorescence were analyzed by flow cytometry (mean ± SD, *p < 0.05, **p < 0.01 vs. 0). KBM5 and KBM5-T315I cells were treated for 96 h with 0.3 μM AKI603, 2 mM NAC or 0.3 μM AKI603 plus 2 mM NAC. The level of DCFH fluorescence were analyzed by flow cytometry (**B**) and cellular senescence was determined by SA-β-gal staining (**C**) (mean ± SD, *p < 0.05, **p < 0.01 vs. AKI). KBM5 and KBM5-T315I cells were treated with 0.3 μM AKI603, 2 mM NAC or 0.3 μM AKI603 plus 2 mM NAC. The growth-inhibitory effects were determined by cell counting assay (96 h) (**D**) or colony formation assay (10 days) (**E**) (mean ± SD, **p < 0.01, ***p < 0.001 vs. AKI). The data are representative of three independent experiments. AKI: AKI603.

Our results demonstrated that AKI603 could induce the production of polyploidy (Supplementary Fig. S3A). We and other previously reported that the level of glycolytic metabolism and the ROS level were significantly increased in the polyploidy cells^16^,^28^,^29^. ROS is considered to be one of the important inducers of senescence^30^,^31^, thus we examined whether ROS production was involved in the occurrence of senescence in KBM5 and KBM5-T315I cells. First, we measured total ROS levels using DCFH (2′,7′-Dichlorofluorescin diacetate) or DHE (Dihydroethidium) fluorescence assay in AKI603-exposed KBM5 and KBM5-T315I cells. As shown in Fig. 4A, after 0.3 μM AKI603 treatment for 96 h, the levels of total ROS fluorescence increased significantly in treated groups of KBM5 and KBM5-T315I cells compared to the control groups. Indeed, AurA knockdown by shRNA could increase the ROS level in KBM5 and K562 cells (Supplementary Fig. S4). Next, we used the ROS scavenger NAC (N-acetyl-L-cysteine) to eliminate cellular ROS^28^. As shown in Fig. 4B, treatment with NAC blocked AKI603-induced ROS accumulation in KBM5 cells (AKI603 + NAC vs AKI603: 838.7 ± 85.4 vs 1378.8 ± 127.1, *p* = 0.007) and KBM5-T315I (AKI603 + NAC vs AKI603: 946.4 ± 92.5 vs 1657.8 ± 105.3, *p* = 0.0035).

Then we analyzed whether ROS accumulation was responsible for AKI603-induced senescence. As shown in Fig. 4C, treatment with NAC significantly reduced the number of SA-β-gal positive cells following AKI603 treatment in both KBM5 cells (AKI603 + NAC vs AKI603: 50.8% ± 4.4% vs 68.1% ± 7%, *p* = 0.0227) and KBM5-T315I (AKI603 + NAC vs AKI603: 60.8% ± 2.6% vs 80.1% ± 4.3%, *p* = 0.0026). NAC application also partially attenuated AKI603-induced reduction in cell numbers of KBM5 (AKI603 + NAC vs AKI603: 16.38 ± 2.93 × 10^4^ vs 5.86 ± 1.65 × 10^4^, *p* = 0.0008) and KBM5-T315I (AKI603 + NAC vs AKI603: 17.19 ± 4.02 × 10^4^ vs 7.94 ± 1.20 × 10^4^, *p* = 0.0044), and in colony formation units of KBM5 (AKI603 + NAC vs AKI603: 8.0 ± 3.0 vs 0, *p* = 0.009) and KBM5-T315I (AKI603 + NAC vs AKI603: 5.7 ± 1.5 vs 0, *p* = 0.003)(Fig. 4D,E). Taken together, these data indicated that AKI603-induced senescence in CML cells was partially through enhancing the ROS generation.

### AKI603 synergistically enhances the effects of imatinib in BCR-ABL-T315I mutant cells

**Figure 5:**
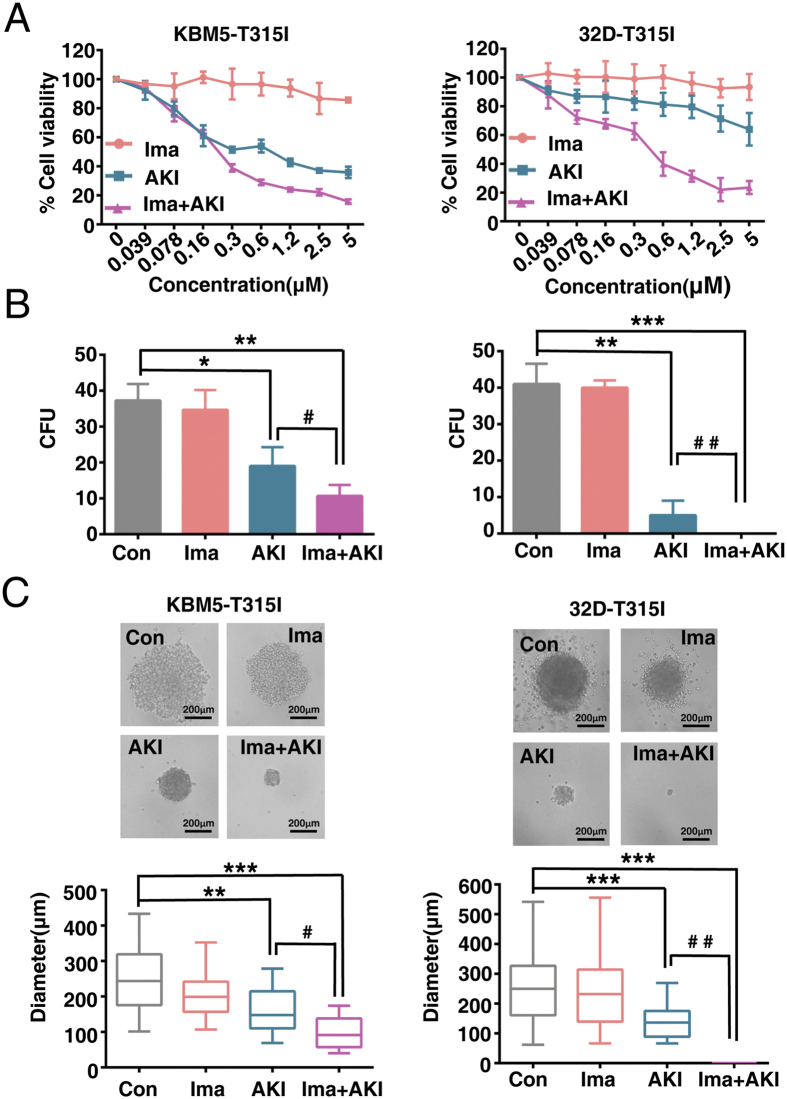
AKI603 and imatinib synergistically inhibit proliferation of cells harboring T315I mutation. (**A**) KBM5-T315I and 32D-T315I cells were treated with indicated concentration of AKI603 for 96 h. The cytostatic activity was measured by MTT assay. Data was the mean of three independent experiments. (**B**) The colony formation of KBM5-T315I and 32D-T315I cells treated with 0.078 μM AKI603, 0.078 μM imatinib or 0.078 μM AKI603 plus 0.078 μM imatinib for 10 days was analyzed. The statistical analysis of the colony formation assay is shown (mean ± SD, *p < 0.05, **p < 0.01, ***p < 0.001 vs. Con; ^#^*p* < 0.05, ^# #^*p* < 0.01vs AKI). (**C**) Representative images are shown and the diameters of colonies were measured. The values from three independent experiments are presented in a box plot graph and the size distribution of the colonies is shown (mean ± SD, **p < 0.01, ***p < 0.001 vs. Con; ^#^*p* < 0.05, ^# #^*p* < 0.01vs AKI). AKI: AKI603.

Anti-tumor therapy with high doses of therapeutic reagents often causes side effects. Therefore, new strategies with lower dose therapeutic reagents are urgently needed. Synergistic analysis was performed to evaluate the combined effects between AKI603 and imatinib in BCR-ABL-T315I mutant cells. The experimental setting of AKI603 and imatinib treatment was combined in a fixed ratio (1:1). The results showed that the combination therapy resulted in a greater growth inhibition of KBM5-T315I and 32D-T315I cells than that was achieved with either AKI603 or imatinib alone (Fig. 5A) and the combination of the two drugs had synergy effect (Supplementary Tables S1 and S2). To confirm these results, a drug concentration of 0.078 μM was used by a colony formation assay. As shown in Fig. 5B, 0.078 μM imatinib did not obviously decrease the number of colony formation in KBM5-T515I (34.7 ± 5.5, *p* = 0.995) and 32D-T315I (40.0 ± 2.0, *p* = 0.78) compared with the controls (KBM5-T315I: 37.3 ± 4.5; 32D-T315I: 41 ± 5.6). AKI603 could inhibit colony formation (KBM5-T315I: 19.0 ± 5.3, *p* = 0.0113; 32D-T315I: 5.0 ± 4.0, *p* = 0.0024) compared with the control groups, whereas the combination (10.7 ± 3.0, *p* = 0.0013 in KBM5-T315I; 0, *p* = 0.000 in 32D-T315I) substantially suppressed colony formation compared with the control groups (Fig. 5B). In addition, the size of the colony formation was measured. The sizes of colony formation did not obviously be decreased at 0.078 μM of imatinib (KBM5-T315I: 203.0 ± 65.2 μm, *p* = 0.1193; 32D-T315I: 243.3 ± 118.9 μm, *p* = 0.997) compared with the control groups (KBM5-T315I: 258.2 ± 77.5 μm; 32D-T315I: 253.6 ± 117.0 μm) (Fig. 5C). 0.078 μM AKI603 decreased the size of colony formation (KBM5-T315I: 162.0 ± 61.6 μm, *p* = 0.0013; 32D-T315I: 136.9 ± 52.7 μm, *p* = 0.0004), whereas the combination significantly suppressed the colony sizes (KBM5-T315I: 95.2 ± 43.5 μm, *P* = 0.00016; 32D-T315I: 0, *p* = 0.000001). These results suggested that the inhibition of AurA by AKI603 provided a potential strategy that overcomes the imatinib resistance in BCR-ABL T315I-mutant cells.

### AKI603 abrogates the growth of xenografted KBM5-T315I cells in nude mice

**Figure 6:**
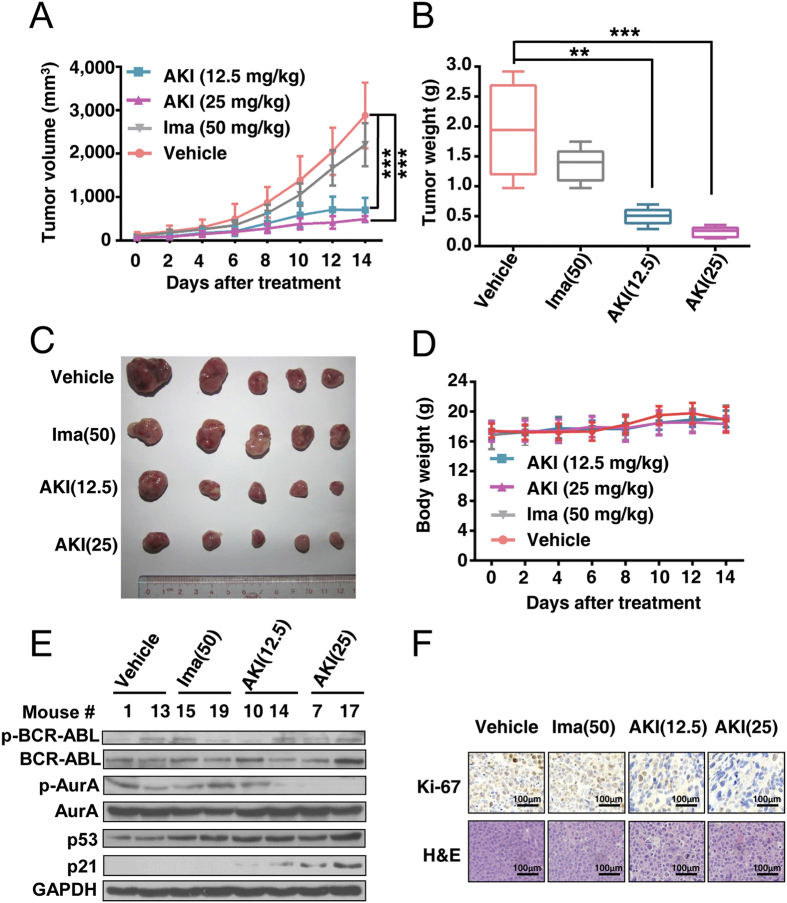
AKI603 abrogates the growth of xenografted KBM5-T315I cells in nude mice. Nude mice bearing KBM5-T315I xenograft tumors were treated with vehicle, imatinib (50 mg/kg/d, treated intragastrically every day), AKI603 (12.5 mg/kg, injected intraperitoneally every 2 days) or AKI603 (25 mg/kg, injected intraperitoneally every 2 days) for 14 days. (**A**) The estimated tumor volume is plotted versus time (n = 5, ***p < 0.001 vs. Vehicle). (**B**) The weights of the dissected tumors were measured (n = 5, **p < 0.01, ***p < 0.001 vs. Vehicle). (**C**) Tumors were removed from 5 mice in each group are shown. (**D**) The body weights were monitored and plotted versus time. (**E**) p-BCR-ABL, BCR-ABL, p-AurA, AurA, p53 and p21 in tumor xenograft tissues were detected by western blot analysis (control group: 1, 13; imatinib-treated group: 15, 19; AKI603-12.5 mg/kg-treated group:10, 14; AKI603-25 mg/kg-treated group: 7, 17). (**F**) Ki-67 was tested by immunohistologic analysis. Hematoxylin and eosin (H&E)-stained sections of the same xenografts are shown.

We further examined the *in vivo* effect of AKI603 on KBM5-T315I cells using the nude mouse xenograft model. As shown in Fig. 6A, the tumor sizes in the AKI603-treated groups (12.5 mg/kg: 699.3 ± 281.2 mm^3^, *p* = 0.00005; 25 mg/kg: 493.2 ± 65.5 mm^3^, *p* = 0.000014) were smaller than that in the vehicle-treated group (2877.3 ± 754.7 mm^3^), indicating that the growth of xenograft tumors was significantly inhibited by AKI603. There was no obvious inhibited effect in imatinib-treated group (2206.5 ± 496.8 mm^3^, *p* = 0.222) compared with the vehicle-treated group. The mean weights of tumors in the treated groups were significantly lower than that in the vehicle-treated group (12.5 mg/kg vs Control: 496.0 ± 145.7 mg vs 1745.2 ± 818.7 mg, *p* = 0.0019; 25 mg/kg vs Control: 234.7 ± 86.5 mg vs 1745.2 ± 818.7 mg, *p* = 0.0003) (Fig. 6B,C). The body weights of the mice were stable, with no significant differences between AKI603 or imatinib-treated and vehicle-treated mice (Fig. 6D). Motor activity and feeding behavior were all normal.

The level of phosphorylation of AurA was significantly decreased in tumor tissues from mice treated with AKI603 than in vehicle treatment, whereas the level of p21 was significantly increased in AKI603-treated groups compared with the vehicle-treated group (Fig. 6E). H&E staining (Hematoxylin-Eosin staining) of tumor slides revealed that cells in the AKI603-treated KBM5-T315I xenograft exhibited greatly enlarged cellular size and these cells were often multinucleated (Fig. 6F). Cell proliferation was reduced in AKI603-treated tumors compared to vehicle-treated tumors by Ki-67 staining (Fig. 6F). Together, the results demonstrated that AKI603 inhibited xenografted KBM5-T315I cells *in vivo*.

## Discussion

Although imatinib has revolutionized the management of CML therapy, drug resistance remains a challenge. Moreover, the prognosis of the patients with imatinib-resistant CML is poor^32^. Extensive efforts have been made to overcome imatinib-resistance. Several groups have developed new generation ATP-competitive BCR-ABL kinase inhibitors, such as nilotinib, dasatinib^33^,^34^. These modified drugs have stronger affinities for the ATP-binding site than imatinib, and thus are more effective for imatinib-resistant patients to some extent. Among many mechanisms proposed to explain the resistance to imatinib, mutation of BCR-ABL (especially T315I mutation) is believed to be the predominant^10^,^32^,^35^. Although these novel inhibitors can effectively inhibit the kinase activity of the mutated BCR-ABL such as E255K and M351T, they had little effect on T315I mutation^36^. We aimed to identify effective therapy against leukemic cells carrying the notorious BCR-ABL-T315I mutation.

We recently reported a novel AurA inhibitor AKI603 that against leukemia cells including BCR-ABL wild-type and T315I mutation. Our findings showed that AKI603 was highly effective in inhibiting the proliferation of leukemic cell lines including CML, AML and ALL (Figs 1 and 2). AKI603 potently inhibited the proliferation and colony formation in CML cells with the T315I mutation (Fig. 2). Interestingly, we found that inhibition of AurA by AKI603 induced senescence partially via enhancing ROS level (Figs 3 and 4). AKI603 and imatinib synergistically inhibited proliferation of cells with BCR-ABL-T315I mutation (Fig. 5). Moreover, AKI603 inhibited the growth of KBM5-T315I cells in nude mouse xenografts (Fig. 6). Thus, AKI603 is effective against CML cells, including those with the T315I mutation *in vitro* and *in vivo*.

Many kinase inhibitors directly bind to the ATP pocket of kinase domain to inhibit the kinase activity^37^. VX-680, is a potent pan-Aurora kinase inhibitor and also inhibits BCR-ABL kinase activity^38^. Different doses of VX-680 have different effects on BCR-ABL. At high doses, VX-680 could inhibit kinase activity of both wild type and T315I mutation BCR-ABL *in vitro* and *in vivo*^18^,^39^. BCR-ABL oncoprotein could suppress cell apoptosis by interactions with the majority of proteins involved in the oncogenic pathways^40^. Some reports showed that low doses of VX-680 and other AurA inhibitor such as MLN8237 inhibited growth of CML cell lines without affecting BCR-ABL activity^39^,^41^,^42^,^43^. Our results demonstrated that low doses of AKI603 did not directly inhibit BCR-ABL kinase activity (Fig. 6) and not significantly induce the apoptosis^23^. These results indicated that inhibition of AurA kinase but not BCR-ABL activity by AKI603 might contribute to the inhibition of tumor growth.

Senescence is an irreversible terminal growth arrest that occurs as a result of cellular stress or DNA damage. A wide variety of anticancer agents have been shown to induce accelerated senescence in tumor cell^44^,^45^. Senescent cells generally display the enlarged and flattened morphology with increased activity of SA-β-galactosidase. Other features of senescence include increased nucleus, vacuolated cellular morphology, high levels of p53, p21, p27 and p16, the DNA damage response (DDR), as well as the senescence-associated secretory phenotype (SASP)^24^. In our study, after treatment with AKI603, CML cells with BCR-ABL-T315I mutation displayed a serial of senescent morphological and functional changes such as enlarged and flattened morphology, increased levels of p21 protein and enhanced SA-β-gal staining as well as imatinib sensitive CML cells (Fig. 3). These results suggested that senescence was a mainly terminal outcome of AurA inhibition in some tumor types.

The role of p53 in the induction of senescence is somewhat controversial. Some studies reported that p53 was necessary for senescence and the onset of senescence was associated with increased levels of p53^46^. Induction of senescence by p53 was associated with the regulation of p53-dependent genes that could participate in cell cycle arrest^47^. However, other evidences showed that p53 could also function to inhibit senescence while promoting cell cycle arrest^48^. In addition, Liu *et al.* recently reported that senescence resulted from inhibition of Aurora kinases was independent of p53^24^. The role of p53 in senescence of different cells responded to different stimulations was different. Our data showed that inhibition AurA with AKI603 induced senescence in both p53 wild type and mutant cells. The level of p21 increased independent of p53 (Fig. 3). This data suggested that p53 was not absolutely required for AKI603-induced senescence.

We and others reported that inhibition AurA kinase by small molecular inhibitors could induce the polyploidization^14^,^16^,^18^. In our study, after treatment with AKI603, the percentage of polyploidy cells was significantly increased. Our previous study showed that the level of glycolytic metabolism was significantly increased in the polyploidy cells induced by AurA inhibitors^16^. Recent study reported that polyploidy cells contained higher levels of ROS due to the higher mitochondrial contents^28^. ROS played an important role in the cellular senescence^30^,^31^. Report also showed that MLN8237 could induce the generation of ROS^49^. We found that the level of ROS was higher in AKI603-treated cells than in control cells. Moreover, knockdown of AurA by shRNA could induce the generation of ROS. These results suggested that AurA inhibited the generation of ROS. Consistent with prior reports^24^, we observed that decreased ROS production and senescence, increased cell viability and cell colony formation after prior treatment of NAC. These results demonstrated that induction of senescence by AKI603 was partially via enhancing ROS level at least.

Combination treatment with a BCR-ABL kinase inhibitor and the other kinase inhibitor could possibly prevent resistance caused by mutations in CML^50^,^51^. Combination with VX-680 or S1451, a novel and highly specific AurA inhibitor, and imatinib caused significantly more cell death than imatinib treatment alone. And combination treatment suppressed development of acquired resistance in KCL-22 cells upon imatinib treatment^41^. Another AurA inhibitor MLN8237 could inhibit growth of CML cells harboring BCR-ABL-T315I mutation or Ba/F3 cells artificially introduced with T315I mutational BCR-ABL. Compared to treatment with either agent alone, the percentage of apoptosis was significantly improved in K562 xenograft tumor treated with nilotinib and MLN8237^42^. In our study, compared to treatment with either agent alone, combination treatment with AKI603 and imatinib also significantly inhibited the growth of BCR-ABL-T315I mutant cells. BCR-ABL is mainly retained within the cytoplasm, where it interacts with the majority of proteins involved in the oncogenic pathway. Examples include Ras/MAPK pathway, JAK/STAT pathway and PI3K/AKT pathway^40^,^52^. One of the most important factor involved in BCR-ABL/ABL signaling was Myc^53^,^54^. ABL could directly activate Myc by phosphorylation on tyrosine 74,^54^. In addition, we and others demonstrated that AurA promoted tumorigenesis and cell survival by overexpression and stabilization of Myc^55^,^56^,^57^. Since Myc is the common downstream protein of both BCR-ABL and AurA, we proposed the combinational effect of AKI603 and imatinib could be caused by inhibition of Myc.

Moreover, in this study, we also investigated the effect of AKI603 on KBM5-T315I cells by using the nude mouse xenograft model. The nude mouse is a useful model for studying the biology and response to therapies of human tumors *in vivo*. In addition, leukemic xenografts implanted subcutaneously into nude mice have played a significant role in testing antitumor activity and cytotoxics of drugs *in vivo*^58^,^59^,^60^,^61^. Our results showed that both 12.5 mg/kg and 25 mg/kg of AKI603 could significantly inhibit the growth of KBM5-T315I xenografts and not affect motor activity and feeding behavior of the nude mice. This indicated that under this administration method and doses, AKI603 did not cause harm to the mice. Xenografts of human tumors or tumor cell lines in nude mice could reproduce the histology and be used to evaluate tumor response to therapy *in vivo*^62^. Our results demonstrated that AKI603 inhibited the growth of KBM5-T315I cells, decreased the activity of AurA and increased the level of p21 in xenografts as well as that *in vitro*. These results offered a novel perspective on the potential effects of AKI603 in CML to some extent *in vivo*.

In summary, we have shown that targeting AurA by AKI603 in imatinib resistant CML cells induced senescence and inhibited tumor growth *in vitro* and *in vivo*. Our results provided new approach for treatment of resistant CML caused by BCR-ABL-T315I mutation with AKI603 in clinical application.

## Materials and Methods

### Chemicals

AKI603, designed and synthesized by our lab^21^, prepared as a 100 mM stock solution in DMSO and stored at −20 °C; 3-(4,5-Dimethylthiazol-2-yl)-2,5-diphenyltetrazolium bromide (MTT), DMSO, Trypan Blue, NAC, CFSE, DCFH and DHE were obtained from Sigma; imatinib (Novartis Pharma Schweiz AG, Switzerland).

### Cell culture

U937, HL-60, Jurkat and K562 were obtained from the American Type Culture Collection (ATCC). NB4 provided by Shanghai Institute of Hematology, Ruijin Hospital. KBM5 and KBM5-T315I cell lines were gifts from Dr. Peng Huang (Texas MD Anderson Cancer Center, Houston, USA). K562/G gifted from Dr. Wen-Lin Huang (Cancer Center, Sun Yat-sen University). 32D cells were obtained from the German Collection of Microorganisms and Cell Cultures (DSMZ, Braunschweig, Germany). All cells were cultured under recommended conditions. Culture media and FBS were from Invitrogen (Carlsbad, CA). All cell lines were authenticated by short tandem repeat profiling.

### Plasmids

MSCV-BCR-ABL-p210 (p210) and MSCV-BCR-ABL-p210-T315I (T315I) were kindly provided by Prof. Justus Duyster (University Medical Center Freiburg, Freiburg, Germany).

### Retrovirus packaging and infection

Stocks of retrovirus were generated by transiently co-transfecting 293-T cell line with p210 or T315I along with the ecotropic packaging plasmids (Addgene) using Lipofectamine 2000 (Invitrogen). Supernatants collected was used to infect the IL-3-dependent murine progenitor cell line 32D. After 48 h, cells were washed once with PBS and cultured in media devoid of IL-3. IL-3-independent 32D cells were propagated and used for biochemical and biological experiments.

### Cell counting assay

About 1 × 10^5^ cells per well were plated in 12-well plates (Corning, Costar, USA). Subsequently, cells were treated with different concentrations AKI603 for 48 h. Then, cell count was determined with Trypan blue exclusion assay.

### MTT assay

1 × 10^4^ cells were seeded in each well of the 96-well plates. Subsequently, cells were treated with different drugs at different concentrations for indicated time. After different treatment, MTT solution was added to each well and cells were incubated at 37 °C for 4 h. The absorbance was finally determined at 490 nm using the microplate reader (BioTek, Vermont, USA).

### CFSE staining assay

Cell proliferation determination was conducted by CFSE probe. Briefly, cells (5 × 10^5^) were seeded and stained with CFSE in 6-well plates according to the manufacturer’s protocol. Then, cells were exposed to a series of concentrations of AKI603 for 48 h. CFSE fluorescence was detected by flow cytometry (Calibur, BD Biosciences, San Diego, CA, USA) and calculated by FlowJo software (Version X; Treestar, Ashland, OR, USA).

### Colony formation assay

Cells were cultured in RPMI 1640 medium supplemented with 0.9% methylcellulose and 10% FBS. The colonies were counted and photographed after 10 days using inverted microscope (Olympus, Tokyo, Japan).

### Cell cycle analysis

The cells were treated with the indicated concentrations of AKI603 for 48 h, collected, and fixed in 70% ice-cold ethanol. After an overnight incubation at 4 °C, the cells were collected by centrifugation, and resuspended in PBS containing 100 μg/mL RNaseA, 50 μg/mL PI and 0.2% Triton X-100. After PI staining, the quantification of the cell-cycle distribution was carried out using a FACS Calibur flow cytometer equipped with FlowJo software.

### ROS assay

ROS generation was analyzed using the fluorescent dyes DCFH and DHE. Briefly, after exposure to AKI603 for 96 h, cells were washed with RPMI 1640 and incubated with one of the fluorescent dyes (10 μM) in RPMI 1640 for 30 min at 37 °C in the dark. Then, cells were washed and analyzed using a flow cytometer at an excitation/emission wavelength of 488/525 nm and 488/610 nm, respectively. The experiments were repeated a minimum of three times.

### SA-β-gal assay

Cells were subjected to SA-β-gal staining using the senescence-associated SA-β-gal Staining Kit (Cell Signaling Technology, MA, USA), according to the protocol. Briefly, cells were washed twice with PBS and fixed with the fixative solution for 15 min at room temperature. Then, cells were washed twice with PBS and incubated overnight at 37 °C with the staining solution before observed using a microscope (Olympus). More than 300 cells per sample were counted to determine the percentage of senescent cells.

### Western blot analysis

Total cellular proteins were isolated with lysis buffer. Equal amounts of protein were subjected to SDS-PAGE and transferred to nitrocellulose membranes. The membranes were blocked and then incubated with p-AurA (Thr288), p-ABL (Tyr245), p53 and p21(Cell Signaling Technology), AurA (Upstate, NewYork, USA), c-ABL and GAPDH (Santa Cruz Biotechnology, Santa Cruz, CA, USA) antibodies. Subsequently, the membranes were incubated with an HRP-conjugated secondary antibody (Cell Signaling Technology) at room temperature for 1 h and were visualized using enhanced chemiluminescence reagents (Sigma), according to the manufacturer’s instruction.

### Bone marrow transduction and transformation

For BCR-ABL transformation assays, murine BM cells were collected from C57BL/6 mice, and transduced with retrovirus, as described^63^. Transduced cells were plated in 6 well cell culture plates for 7 days without cytokines and then used for further experiments.

### Tumor growth in xenografts

3 × 10^7^ of KBM5-T315I cells were injected into the flanks of female BALB/c nude mice. Tumors were measured every other day with use of calipers. Tumor volumes were calculated by the following formula: *A* × *B*^2^/2, where *A* is the greatest diameter and *B* is the diameter perpendicular to *A*. Other indicators of general health, such as body weight, feeding behavior, and motor activity, of each animal were also monitored. 6 days after subcutaneous inoculation, when tumors were palpable (50~150 mm^3^), mice were randomized to receive treatment with vehicle (50% PEG300 in 50 mM PBS), AKI603 (12.5 mg/kg or 25 mg/kg, injected intraperitoneally every 2 days) and imatinib (50 mg/kg, treated intragastrically every day) for 2 weeks. The animals were then euthanized, and tumor xenografts were immediately removed, weighed, stored, and fixed.

### Ethics statement

The methods were carried out in accordance with the Guide for the Care and Use of Laboratory Animals (2011). The study protocol was approved by the Animal Ethical and Welfare Committee (AEWC) of Sun Yat-sen University (The Approved No. is IACUC-F3-14-1201).

### Statistical analysis

The data are representative of three independent experiments. Data were presented as mean ± SD. Statistical analysis was performed using Prism 6 (GraphPad Software, Inc.) and SPSS v. 16.0 (SPSS, Inc.). The unpaired two-tailed Student’s t test was used to perform statistical comparison between two groups. The ANOVA test was used for multiple comparisons. The Kruskal-Wallis test, followed by Dunn’s Multiple Comparison test, was used to perform statistical comparison for colonies size distribution. *p* < 0.05 was considered statistically.

## Additional Information

**How to cite this article**: Wang, L.-X. *et al.* Aurora A Kinase Inhibitor AKI603 Induces Cellular Senescence in Chronic Myeloid Leukemia Cells Harboring T315I Mutation. *Sci. Rep.* **6**, 35533; doi: 10.1038/srep35533 (2016).

**Publisher’s note**: Springer Nature remains neutral with regard to jurisdictional claims in published maps and institutional affiliations.

## Supplementary Material

Supplementary Information

## References

[b1] NCCN. NCCN clinical practice guidelines in oncology. *NCCN Chronic Myelogenous Leukemia Guidelines* Vers 1, NCCN (2015).

[b2] Faderl S. *et al.* The biology of chronic myeloid leukemia. N Engl J Med. 341, 164–172 (1999). 10403855 10.1056/NEJM199907153410306

[b3] Melo J. V. & Barnes D. J. Chronic myeloid leukaemia as a model of disease evolution in human cancer. Nat Rev Cancer. 7, 441–453 (2007). 17522713 10.1038/nrc2147

[b4] Gesbert F., Sellers W. R., Signoretti S., Loda M. & Griffin J. D. BCR/ABL regulates expression of the cyclin-dependent kinase inhibitor p27Kip1 through the phosphatidylinositol 3-Kinase/AKT pathway. J Biol Chem. 275, 39223–39230 (2000). 11010972 10.1074/jbc.M007291200

[b5] Lugo T. G., Pendergast A. M., Muller A. J. & Witte O. N. Tyrosine kinase activity and transformation potency of bcr-abl oncogene products. Science. 247, 1079–1082 (1990). 2408149 10.1126/science.2408149

[b6] Danial N. N. & Rothman P. JAK-STAT signaling activated by Abl oncogenes. Oncogene. 19, 2523–2531 (2000). 10851051 10.1038/sj.onc.1203484

[b7] Druker B. J. *et al.* Efficacy and safety of a specific inhibitor of the BCR-ABL tyrosine kinase in chronic myeloid leukemia. N Engl J Med. 344, 1031–1037 (2001). 11287972 10.1056/NEJM200104053441401

[b8] O’Hare T., Zabriskie M. S., Eiring A. M. & Deininger M. W. Pushing the limits of targeted therapy in chronic myeloid leukaemia. Nat Rev Cancer. 12, 513–526 (2012). 22825216 10.1038/nrc3317

[b9] Zabriskie M. S. *et al.* BCR-ABL1 compound mutations combining key kinase domain positions confer clinical resistance to ponatinib in Ph chromosome-positive leukemia. Cancer cell. 26, 428–442 (2014). 25132497 10.1016/j.ccr.2014.07.006 PMC4160372

[b10] Apperley J. F. Part I: mechanisms of resistance to imatinib in chronic myeloid leukaemia. Lancet Oncol. 8, 1018–1029 (2007). 17976612 10.1016/S1470-2045(07)70342-X

[b11] O’Hare T. *et al.* AP24534, a pan-BCR-ABL inhibitor for chronic myeloid leukemia, potently inhibits the T315I mutant and overcomes mutation-based resistance. Cancer cell. 16, 401–412 (2009). 19878872 10.1016/j.ccr.2009.09.028 PMC2804470

[b12] Ikezoe T. *et al.* A novel treatment strategy targeting Aurora kinases in acute myelogenous leukemia. Mol Cancer Ther. 6, 1851–1857 (2007). 17541033 10.1158/1535-7163.MCT-07-0067

[b13] Kelly K. R. *et al.* Phase I study of MLN8237–investigational Aurora A kinase inhibitor–in relapsed/refractory multiple myeloma, non-Hodgkin lymphoma and chronic lymphocytic leukemia. Invest New Drugs. 32, 489–499 (2014). 24352795 10.1007/s10637-013-0050-9 PMC4045308

[b14] Harrington E. A. *et al.* VX-680, a potent and selective small-molecule inhibitor of the Aurora kinases, suppresses tumor growth *in vivo*. Nat Med. 10, 262–267 (2004). 14981513 10.1038/nm1003

[b15] Goldberg S. L. *et al.* An exploratory phase 2 study of investigational Aurora A kinase inhibitor alisertib (MLN8237) in acute myelogenous leukemia and myelodysplastic syndromes. Leuk Res Rep. 3, 58–61 (2014). 25068104 10.1016/j.lrr.2014.06.003 PMC4110881

[b16] Liu L. L. *et al.* Inhibition of mTOR pathway sensitizes acute myeloid leukemia cells to aurora inhibitors by suppression of glycolytic metabolism. Mol Cancer Res. 11, 1326–1336 (2013). 24008673 10.1158/1541-7786.MCR-13-0172

[b17] Cammareri P. *et al.* Aurora-a is essential for the tumorigenic capacity and chemoresistance of colorectal cancer stem cells. Cancer Res. 70, 4655–4665 (2010). 20460511 10.1158/0008-5472.CAN-09-3953

[b18] Giles F. J. *et al.* MK-0457, a novel kinase inhibitor, is active in patients with chronic myeloid leukemia or acute lymphocytic leukemia with the T315I BCR-ABL mutation. Blood. 109, 500–502 (2007). 16990603 10.1182/blood-2006-05-025049

[b19] Akahane D., Tauchi T., Okabe S., Nunoda K. & Ohyashiki K. Activity of a novel Aurora kinase inhibitor against the T315I mutant form of BCR-ABL: *in vitro* and *in vivo* studies. Cancer Sci. 99, 1251–1257 (2008). 18429956 10.1111/j.1349-7006.2008.00810.x PMC11158598

[b20] Carter T. A. *et al.* Inhibition of drug-resistant mutants of ABL, KIT, and EGF receptor kinases. Proc Natl Acad Sci USA. 102, 11011–11016 (2005). 16046538 10.1073/pnas.0504952102 PMC1180625

[b21] Zheng F. M. *et al.* A novel small molecule aurora kinase inhibitor attenuates breast tumor-initiating cells and overcomes drug resistance. Mol Cancer Ther. 13, 1991–2003 (2014). 24899685 10.1158/1535-7163.MCT-13-1029

[b22] Pan X. N. *et al.* Inhibition of c-Myc overcomes cytotoxic drug resistance in acute myeloid leukemia cells by promoting differentiation. Plos One. 9, e105381; 10.1371/journal.pone.0105381 (2014). 25127121 10.1371/journal.pone.0105381 PMC4134294

[b23] Long Z. J. *et al.* A novel compound against oncogenic Aurora kinase A overcomes imatinib resistance in chronic myeloid leukemia cells. Int J Oncol. 46, 2488–2496 (2015). 25872528 10.3892/ijo.2015.2960

[b24] Liu Y. *et al.* Targeting aurora kinases limits tumour growth through DNA damage-mediated senescence and blockade of NF-kappaB impairs this drug-induced senescence. EMBO Mol Med. 5, 149–166 (2013). 23180582 10.1002/emmm.201201378 PMC3569660

[b25] Kim H. J., Cho J. H., Quan H. & Kim J. R. Down-regulation of Aurora B kinase induces cellular senescence in human fibroblasts and endothelial cells through a p53-dependent pathway. FEBS lett. 585, 3569–3576 (2011). 22024481 10.1016/j.febslet.2011.10.022

[b26] Brusa G. *et al.* p53 loss of function enhances genomic instability and accelerates clonal evolution of murine myeloid progenitors expressing the p(210)BCR-ABL tyrosine kinase. Haematologica. 88, 622–630 (2003). 12801837

[b27] Sen S., Takahashi R., Rani S., Freireich E. J. & Stass S. A. Expression of differentially phosphorylated Rb and mutant p53 proteins in myeloid leukemia cell lines. Leuk Res. 17, 639–647 (1993). 8355507 10.1016/0145-2126(93)90068-v

[b28] Roh M., van der Meer R. & Abdulkadir S. A. Tumorigenic polyploid cells contain elevated ROS and ARE selectively targeted by antioxidant treatment. J Cell Physiol. 227, 801–812 (2012). 21503880 10.1002/jcp.22793 PMC3156849

[b29] McCrann D. J., Yang D., Chen H., Carroll S. & Ravid K. Upregulation of Nox4 in the aging vasculature and its association with smooth muscle cell polyploidy. Cell Cycle. 8, 902–908 (2009). 19221493 10.4161/cc.8.6.7900 PMC2744814

[b30] Macip S. *et al.* Inhibition of p21-mediated ROS accumulation can rescue p21-induced senescence. EMBO J. 21, 2180–2188 (2002). 11980715 10.1093/emboj/21.9.2180 PMC125979

[b31] Campisi J. & d’Adda di Fagagna F. Cellular senescence: when bad things happen to good cells. Nat Rev Mol Cell Biol. 8, 729–740 (2007). 17667954 10.1038/nrm2233

[b32] Kantarjian H. M., Talpaz M., Giles F., O’Brien S. & Cortes J. New insights into the pathophysiology of chronic myeloid leukemia and imatinib resistance. Ann Intern Med. 145, 913–923 (2006). 17179059 10.7326/0003-4819-145-12-200612190-00008

[b33] Kaur P. *et al.* Nilotinib treatment in mouse models of P190 Bcr/Abl lymphoblastic leukemia. Mol Cancer. 6, 67 (2007). 17958915 10.1186/1476-4598-6-67 PMC2169263

[b34] Talpaz M. *et al.* Dasatinib in imatinib-resistant Philadelphia chromosome-positive leukemias. N Engl J Med. 354, 2531–2541 (2006). 16775234 10.1056/NEJMoa055229

[b35] Shah N. P. Loss of response to imatinib: mechanisms and management. *Hematology-Am Soc Hemat*. 1, 183–187 (2005). 10.1182/asheducation-2005.1.183 16304378

[b36] Akard L. P. Second-generation BCR-ABL kinase inhibitors in CML. N Engl J Med. 363, 1672–1673 (2010). 10.1056/NEJMc1007927 20973144

[b37] Cohen P. & Alessi D. R. Kinase drug discovery–what’s next in the field? ACS Chem Biol. 8, 96–104 (2013). 23276252 10.1021/cb300610s PMC4208300

[b38] Cheetham G. M., Charlton P. A., Golec J. M. & Pollard J. R. Structural basis for potent inhibition of the Aurora kinases and a T315I multi-drug resistant mutant form of Abl kinase by VX-680. Cancer Lett. 251, 323–329 (2007). 17240048 10.1016/j.canlet.2006.12.004

[b39] Donato N. J. *et al.* Targets and effectors of the cellular response to aurora kinase inhibitor MK-0457 (VX-680) in imatinib sensitive and resistant chronic myelogenous leukemia. Biochem Pharmacol. 79, 688–697 (2010). 19874801 10.1016/j.bcp.2009.10.009

[b40] Cilloni D. & Saglio G. Molecular pathways: BCR-ABL. Clin Cancer Res. 18, 930–937 (2012). 22156549 10.1158/1078-0432.CCR-10-1613

[b41] Yuan H. *et al.* Overcoming CML acquired resistance by specific inhibition of Aurora A kinase in the KCL-22 cell model. Carcinogenesis. 33, 285–293 (2012). 22116466 10.1093/carcin/bgr278 PMC3271265

[b42] Kelly K. R. *et al.* The novel Aurora A kinase inhibitor MLN8237 is active in resistant chronic myeloid leukaemia and significantly increases the efficacy of nilotinib. J Cell Mol Med. 15, 2057–2070 (2011). 21091633 10.1111/j.1582-4934.2010.01218.x PMC4394217

[b43] Fiskus W. *et al.* Cotreatment with vorinostat enhances activity of MK-0457 (VX-680) against acute and chronic myelogenous leukemia cells. Clin Cancer Res. 14, 6106–6115 (2008). 18829489 10.1158/1078-0432.CCR-08-0721 PMC2665710

[b44] Roninson I. B. Tumor cell senescence in cancer treatment. Cancer research. 63, 2705–2715 (2003). 12782571

[b45] Chang B. D. *et al.* A senescence-like phenotype distinguishes tumor cells that undergo terminal proliferation arrest after exposure to anticancer agents. Cancer Res. 59, 3761–3767 (1999). 10446993

[b46] Huck J. J. *et al.* MLN8054, an inhibitor of Aurora A kinase, induces senescence in human tumor cells both *in vitro* and *in vivo*. Mol Cancer Res. 8, 373–384 (2010). 20197380 10.1158/1541-7786.MCR-09-0300

[b47] Vigneron A. & Vousden K. H. p53, ROS and senescence in the control of aging. Aging (Albany NY). 2, 471–474 (2010). 20729567 10.18632/aging.100189 PMC2954038

[b48] Demidenko Z. N., Korotchkina L. G., Gudkov A. V. & Blagosklonny M. V. Paradoxical suppression of cellular senescence by p53. Proc Natl Acad Sci USA. 107, 9660–9664 (2010). 20457898 10.1073/pnas.1002298107 PMC2906905

[b49] Niu N. K. *et al.* Pro-apoptotic and pro-autophagic effects of the Aurora kinase A inhibitor alisertib (MLN8237) on human osteosarcoma U-2 OS and MG-63 cells through the activation of mitochondria-mediated pathway and inhibition of p38 MAPK/PI3K/Akt/mTOR signaling pathway. Drug Des Dev Ther. 9, 1555–1584 (2015). 10.2147/DDDT.S74197 PMC4362906 25792811

[b50] O’Hare T., Eide C. A. & Deininger M. W. Bcr-Abl kinase domain mutations, drug resistance, and the road to a cure for chronic myeloid leukemia. Blood. 110, 2242–2249 (2007). 17496200 10.1182/blood-2007-03-066936

[b51] Carter B. Z. *et al.* Combined targeting of BCL-2 and BCR-ABL tyrosine kinase eradicates chronic myeloid leukemia stem cells. Sci Transl Med. 8, 355ra117, doi: 10.1126/scitranslmed.aag1180 (2016). PMC5111086 27605552

[b52] Melo J. V. & Deininger M. W. Biology of chronic myelogenous leukemia–signaling pathways of initiation and transformation. HematolOncol Clin N. 18, 545–568 (2004). 10.1016/j.hoc.2004.03.008 15271392

[b53] Sawyers C. L., Callahan W. & Witte O. N. Dominant negative MYC blocks transformation by ABL oncogenes. Cell. 70, 901–910 (1992). 1525828 10.1016/0092-8674(92)90241-4

[b54] Sanchez-Arevalo Lobo V. J. *et al.* Dual regulation of Myc by Abl. Oncogene. 32, 5261–5271 (2013). 23318434 10.1038/onc.2012.621 PMC3914638

[b55] Zheng F. *et al.* Nuclear AURKA acquires kinase-independent transactivating function to enhance breast cancer stem cell phenotype. Nat Commun. 7, 10180 (2016). 26782714 10.1038/ncomms10180 PMC4735655

[b56] Lee J. K. *et al.* N-Myc Drives Neuroendocrine Prostate Cancer Initiated from Human Prostate Epithelial Cells. Cancer Cell. 29, 536–547 (2016). 27050099 10.1016/j.ccell.2016.03.001 PMC4829466

[b57] Dauch D. *et al.* A MYC-aurora kinase A protein complex represents an actionable drug target in p53-altered liver cancer. Nat Med. 22, 744–753 (2016). 27213815 10.1038/nm.4107

[b58] Shi X. *et al.* Triptolide inhibits Bcr-Abl transcription and induces apoptosis in STI571-resistant chronic myelogenous leukemia cells harboring T315I mutation. ClinCancer Res. 15, 1686–1697 (2009). 10.1158/1078-0432.CCR-08-2141 19240172

[b59] Wu Y. *et al.* Cyclin-dependent kinase 7/9 inhibitor SNS-032 abrogates FIP1-like-1 platelet-derived growth factor receptor alpha and bcr-abl oncogene addiction in malignant hematologic cells. Clin Cancer Res. 18, 1966–1978 (2012). 22447844 10.1158/1078-0432.CCR-11-1971

[b60] Shi X. *et al.* Gambogic acid induces apoptosis in imatinib-resistant chronic myeloid leukemia cells via inducing proteasome inhibition and caspase-dependent Bcr-Abl downregulation. Clin Cancer Res. 20, 151–163 (2014). 24334603 10.1158/1078-0432.CCR-13-1063 PMC3938960

[b61] Valsasina B. *et al.* NMS-P937, an orally available, specific small-molecule polo-like kinase 1 inhibitor with antitumor activity in solid and hematologic malignancies. Mol Cancer Ther. 11, 1006–1016 (2012). 22319201 10.1158/1535-7163.MCT-11-0765

[b62] Kerbel R. S. Human tumor xenografts as predictive preclinical models for anticancer drug activity in humans - Better than commonly perceived - But they can be improved. Cancer Biol Ther. 2, S134–S139 (2003). 14508091

[b63] Miething C. *et al.* The Bcr-Abl mutations T315I and Y253H do not confer a growth advantage in the absence of imatinib. Leukemia. 20, 650–657 doi: 10.1038/sj.leu.2404151 (2006). 16482207

