# Supplementary material for: Aurora A Kinase Inhibitor AKI603 Induces Cellular Senescence in Chronic Myeloid Leukemia Cells Harboring T315I Mutation
Source: Sci Rep. 2016 Nov 8;6:35533. doi: 10.1038/srep35533 (PMC5099696; doi:10.1038/srep35533)
Supplement: Supplementary Information [file srep35533-s1.pdf]

Aurora A Kinase Inhibitor AKI603 Induces Cellular Senescence in Chronic Myeloid Leukemia Cells Harboring T315I Mutation

Le-Xun Wang[1,2,*], Jun-Dan Wang[1,*], Jia-Jie Chen[1,*], Bing Long[1], Ling-Ling Liu[1], Xi-Xiang Tu[3], Yu Luo[4], Yuan Hu[1], Dong-Jun Lin[1], Gui Lu[4], Zi-Jie Long[1] & Quentin Liu[1,3,5]

[1]Department of Hematology, The Third Affiliated Hospital, Sun Yat-sen University, 600 Tianhe Road, Guangzhou 510630, China; Institute of Hematology, Sun Yat-sen University, Guangzhou 510630, China;

[2]Department of Cardiac Surgery II, The First Affiliated Hospital, Sun Yat-sen University, 58 Zhongshan 2 Road, Guangzhou 510080, China.

[3]Institute of Cancer Stem Cell, Dalian Medical University, 9 West Section, Lvshun South Road, Dalian 116044, China;

[4]Institute of Medicinal Chemistry, School of Pharmaceutical Sciences, Sun Yat-sen University, 132 Waihuan Road East, Guangzhou 510006, China;

[5]Sun Yat-sen University Cancer Center; State Key Laboratory of Oncology in South China; Collaborative Innovation Center of Cancer Medicine, 651 Dongfeng East Road, Guangzhou 510060, China;

*These authors have contributed equally to this work.

Correspondence authors: Quentin Liu, Department of Hematology, The Third

Affiliated Hospital, Sun Yat-sen University, 600 Tianhe Road, Guangzhou 510630, China; Tel: (+86)-20-85253601; Fax: (+86)-20-85252874; E-mail: liuq9@mail.sysu.edu.cn; Zi-Jie Long, Department of Hematology, Third Affiliated Hospital, Sun Yat-sen University, 600 Tianhe Road, Guangzhou 510630, China; Tel: (+86)-20-85253601; E-mail: longzij@mail.sysu.edu.cn; Gui Lu, Institute of Medicinal Chemistry, School of Pharmaceutical Sciences, Sun Yat-sen University, 132 Waihuan Road East, Guangzhou 510006, China; Tel: (+86)-20-39943048; E-mail: lugui@mail.sysu.edu.cn.

Inventory of Supplemental Information

a) Supplemental Experimental Procedures

b) Supplemental Tables

c) Supplemental Figures

d) Supplemental Figure Legends

Supplemental Experimental Procedures

Plasmid and shRNA stable cell lines

Knockdown of human AurA was performed with the specific shRNA of AurA (shAurA) and the nontarget shRNA (shC) delivered to KBM5 and K562 by a lentiviral system purchased from Sigma according to the instruction manual.

Apoptosis analysis

Cells were treated with the indicated concentration of AKI603 for 48 h or 96 h， collected and resuspended in the binding buffer. Annexin-V and PI was added to the cells according to the protocol by the Annexin V/PI apoptosis detection kit (BD Biosciences). The cells were then incubated for 15 min in the dark and subjected to flow cytometry analysis.

Hematoxylin and Eosin (H&E) Staining

The tissues were transferred through a series of graded alcohol followed by paraffin embedding. The tissue was sectioned at 5 μm thickness and stained with conventional hematoxylin and eosin (H&E) staining. The stained sections were observed by light microscope (Olympus).

Immunohistochemistry (IHC)

Ki-67 was detected in xenografts by IHC. After ethanol exposure and hydration, the sections were rinsed in PBS, quenched for 10 min in methanol containing 3% H_2O_2, and incubated for 15 min in blocking solution (PBS containing 2% goat serum, 0.2% milk, and 0.1% Triton X-100), followed by incubation overnight in primary antibodies against Ki-67 (Sigma), diluted 1:50 in blocking solution. After washing

with PBS, the sections were incubated for 60 min with the secondary antibody (Thermo Fisher Scientific). DAB substrate kit for peroxidase was then used to stain sections as described in the manufacturer's instructions (Sigma). The sections were counterstained with hematoxylin and were observed by light microscope (Olympus).

Supplementary Table S1: Synergy analysis of Imatinib/AKI603 in KBM5-T315I cells using Calcusyn software.

Imatinib (μM)	AKI603 (μM)	Fa	CI
0.039	0.039	0.0592	5.970
0.078	0.078	0.2416	0.662
0.16	0.16	0.3735	0.436
0.3	0.3	0.6112	0.157
0.6	0.6	0.7105	0.142
1.2	1.2	0.7590	0.183
2.5	2.5	0.7784	0.314
5	5	0.8422	0.300

Combination index (CI) value was generated using CalcuSyn software (Biosoft, Ferguson, MO) for detecting the combination effects of Imatinib and AKI603. A value of CI< 0.9 indicates synergism.

Supplementary Table S2: Synergy analysis of Imatinib/AKI603 in 32D-T315I cells using Calcusyn software.

Imatinib (μM)	AKI603 (μM)	Fa	CI
0.039	0.039	0.1201	0.388
0.078	0.078	0.2768	0.032
0.16	0.16	0.2216	0.159
0.3	0.3	0.2341	0.254
0.6	0.6	0.2616	0.322
1.2	1.2	0.3058	0.329
2.5	2.5	0.4594	0.090
5	5	0.6046	0.030

A value of CI< 0.9 indicates synergism.

Supplementary Figures

Supplementary Figure S1

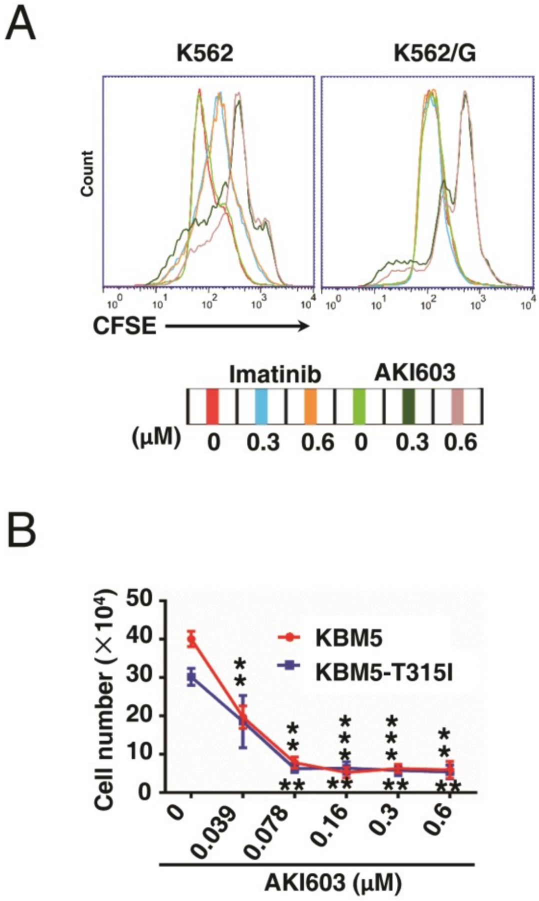

Supplementary Figure S2

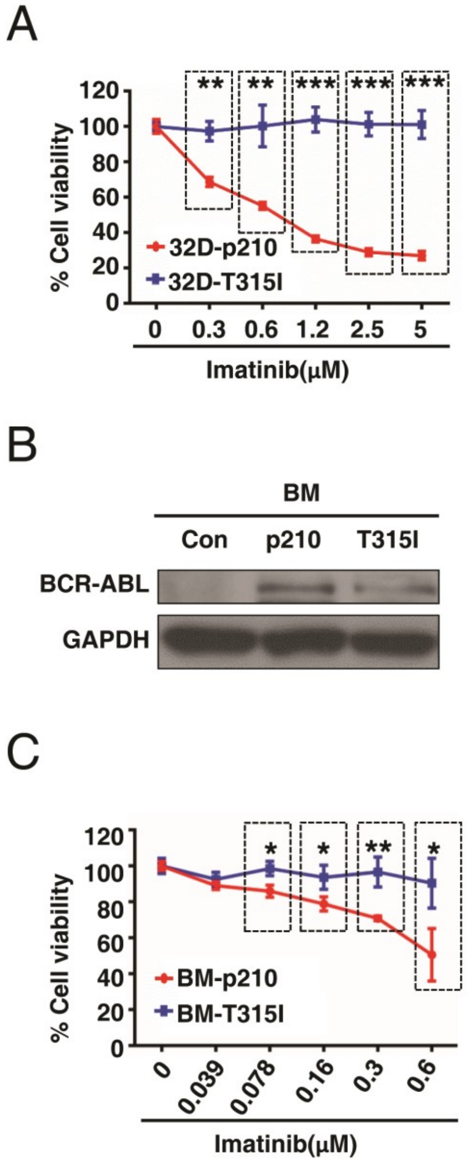

Supplementary Figure S3

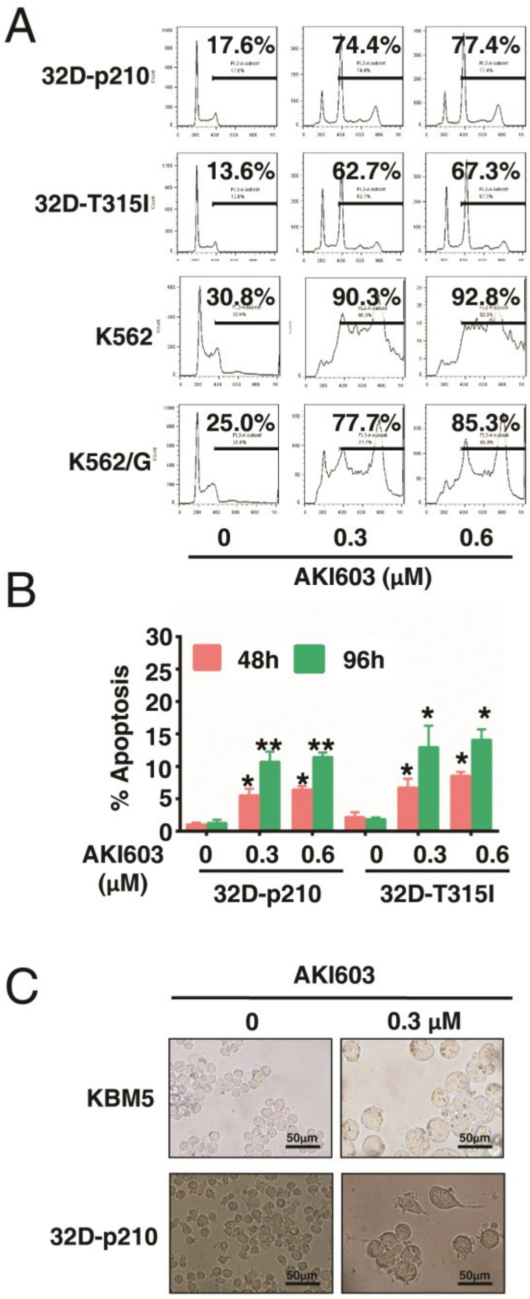

Supplementary Figure S4

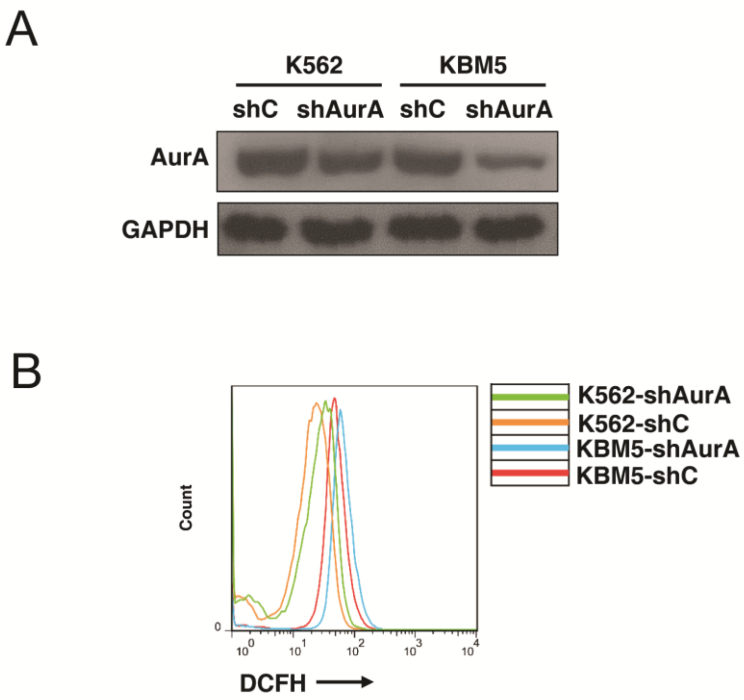

Supplemental Figure Legends

Supplementary Figure S1. AKI603 inhibits the proliferation of imatinib resistant cells.

(A) K562 and K562/G cells were stained by CFSE probe and treated with various concentrations of AKI603 or imatinib for 48 h. The levels of CFSE fluorescence were analyzed by flow cytometry. The data are representative of three independent experiments. (B) KBM5 and KBM5-T315I cells were treated with various concentrations of AKI603 for 48 h. Cell counting assay was performed (mean ± SD, **: $p < 0.01$, ***: $p < 0.001$ vs. 0).

Supplementary Figure S2. Cells with BCR-ABL-T315I are resistant to imatinib.

(A) 32D-p210 and 32D-T315I cells were treated with different doses of imatinib for 48 h and subjected to MTT assay. The mean values from three independent experiments are presented (mean ± SD, **: $p < 0.01$, ***: $p < 0.001$). (B) After infection with p210 or p210-T315I retrovirus, BM cells were cultured for 7 days without cytokines. The lysates were subjected for western blot analysis of BCR-ABL. (C) BM-p210 and BM-T315I cells were treated with different doses of imatinib for 96 h and subjected to MTT assay. The data are representative of three independent experiments (mean ± SD, *: $p < 0.05$, **: $p < 0.01$).

Supplementary Figure S3. AKI603 induces polyploidization but does not obvious apoptosis in imatinib resistant cells.

(A) K562, K562/G, 32D-p210 and 32D-T315I cells were treated with various concentrations of AKI603 for 48 h and cell cycle was analyzed by flow cytometry. The data are representative of three independent experiments. (B) 32D-p210 and 32D-T315I cells were treated with various concentrations of AKI603 for 48 h and 96 h, and the apoptosis was analyzed by flow cytometry (mean ± SD, *: $p < 0.05$, **: $p < 0.01$ vs. 0). (C) KBM5 and 32D-p210 cells were treated with 0.3 μM AKI603 for 96 h and the change in morphology was captured with a microscope.

Supplementary Figure S4. Knockdown of AurA increases the ROS level in KBM5 and K562 cells.

(A) KBM5 and K562 cells were delivered with shAurA and shC by lentiviral system. After 48 h, the lysates were subjected for western blot analysis of AurA and GAPDH expression. (B) The levels of DCFH fluorescence were analyzed by flow cytometry. The data are representative of three independent experiments.
